# Black and Green Tea as Well as Specialty Teas Increase Osteoblast Mineralization with Varying Effectiveness

**DOI:** 10.1089/jmf.2020.0139

**Published:** 2021-08-17

**Authors:** Michael D. McAlpine, William Gittings, Adam J. MacNeil, Wendy E. Ward

**Affiliations:** ^1^ Department of Kinesiology, Faculty of Applied Health Sciences, Brock University, St. Catharines, Ontario, Canada.; ^2^ Center for Bone and Muscle Health, Brock University, St. Catharines, Ontario, Canada.; ^3^ Department of Health Sciences, Faculty of Applied Health Sciences, Brock University, St. Catharines, Ontario, Canada.

**Keywords:** antioxidant, bone, mineral, osteoblast, polyphenol, tea

## Abstract

Many human studies suggest a benefit of tea consumption on bone health. The study objective was to compare the ability of different tea types to promote mineralization. Saos-2 cells underwent mineralization (5 days) in the presence of tea (white: WT, green: GT, black: BT, green rooibos: GR, or red rooibos: RR; 1 μg/mL of polyphenols) or control. Total polyphenol content (TPC, Folin-Ciocalteu's reagent), antioxidant capacity (2,2-diphenyl-1-picrylhydrazyl [DPPH] scavenging), mineralization (Alizarin Red staining), gene expression quantitative reverse transcription PCR (RT-qPCR), and cell activity (3-[4,5-dimethylthiazol-2-yl]-2,5-diphenyltetrazolium bromide assay) were determined. TPC was highest in GT and BT. The ability of each tea to inhibit DPPH also differed (WT, GT > RR) after normalizing for polyphenol quantity. Each tea increased mineralization and differences were observed among types (GT/BT/GR/RR > WT, GT = BT = GR, RR > BT/GT). mRNA expression of alkaline phosphatase (*ALP*) and ectonucleotide pyrophosphatase/phosphodiesterase (*NPP1*) remained unchanged, whereas osteopontin (*OPN*) and sclerostin (*SOST*) were reduced in cells treated with tea, regardless of type. At 24- and 48-h postexposure to tea, cell activity was greater in cells receiving any of the teas compared with vehicle control. Supplementation increased mineralization regardless of tea type with both rooibos teas and black tea stimulating greater mineralization than WT, whereas green tea is similar to the others. While future study is needed to confirm *in vivo* effects, the results suggest that consuming any of the teas studied may benefit bone health.

## Introduction

The *Camellia sinensis* plant is the source of “true teas,” including white, green, and black teas. The tea type produced depends on the level of processing and this, in turn, influences the content and profile of polyphenols that define the unique taste characteristics. Many of the documented health benefits of tea are often attributed to its polyphenols and their ability to function as antioxidants.^1^ Higher consumption of more common teas—black and green—have been associated with reduced incidence of cancer, stroke, and cardiovascular disease,^2–4^ and higher bone mineral density (BMD).^5,6^ Although not made from *C. sinensis*, herbal teas—prepared from roots, leaves, seeds, flowers, or bark of plants—also contain an abundance of unique polyphenols, are often caffeine free, and have commonly been used in traditional medicine.^7^ As of 2015, the Canadian tea market has seen increases in tea consumption with a third of this consumption being regular teas (green and black) and the remaining two thirds being specialty teas (rooibos, white, chai, and flavored teas).^8^ Furthermore, the market is likely to shift even more as younger generations have been shown to prefer specialty teas over regular teas.^8^ Rooibos is a specialty tea that has increased in popularity.^9^ Derived from the South African *Aspalathus linearis* plant, rooibos tea can be classified as either red or green rooibos depending on the level of oxidation during processing; red rooibos is oxidized whereas green is not. Of the teas originating from the *C. sinensis* plant, white tea is the least processed and oxidized with a refreshing mild taste.

Other than the aforementioned health effects associated with consumption of green and black teas, tea polyphenols may also support bone health. During childhood and early adolescence, BMD increases rapidly and is traditionally followed by a period where there is no net change. After this period, a slow reduction in BMD begins, which is often exacerbated in females due to menopause and estrogen loss ultimately increasing osteoclast activity and of bone resorption.^10^ Eventually, this may lead to osteoporosis, or a general weakening of the bones, and an increased risk of fracture. The effects of tea and its specific polyphenols on both osteoblasts and osteoclasts have been studied using cell models.^11–19^ The majority of studies using osteoblasts have studied the effects of green or black teas and have consistently shown increases in mineralization in response to tea. More specifically black, green, and rooibos (red and green) teas have been shown to increase mineralization at concentrations likely attainable through diet.^11–13^ As well, individual polyphenols and extracts derived from black, green, and rooibos teas have been studied and demonstrate a capacity to increase mineralization; however, the concentrations required to demonstrate these effects are usually at a level that would require supplementation.^14–16^ While studies in osteoclasts are limited, previously published studies have shown that black, green, or green rooibos teas elicit a decrease in activity.^17–19^ The majority of literature has investigated green and black teas. Based on current trends of tea consumption in the Canadian market, information regarding the influence of specialty teas on health, including bone health, may further increase interest among consumers.

It is unclear if certain tea types are more effective at promoting mineralization than others. Previously, only one study has compared the effects of a normalized concentration (1 μg/mL of gallic acid equivalents [GAE]) of green, black, and green rooibos tea on osteoblast cell mineralization after 7 days.^11^ Similar to other cell studies investigating individual tea types,^12,13,20^ findings demonstrated increased osteoblast mineralization independent of tea type, with minimal differences between the tea types, despite differing polyphenol profiles. Thus, the quantity rather than the profile of polyphenols in tea may be more important to increased mineralization effects. Of note was that this previous study investigated green rooibos rather than red rooibos, although red rooibos is more commonly consumed. Given the interest of consumers in specialty teas, beyond black and green tea, the objective of this study was to measure and directly compare the ability of different tea types, including black and green tea as well as specialty teas (white, green rooibos, red rooibos) to promote mineralization by osteoblasts. It was hypothesized that all tea types studied would increase osteoblast mineralization, but there would be minimal differences between the different tea types as previous studies reported similar effectiveness to increase mineralization despite differing polyphenol profiles.

## Materials and Methods

Use of Saos-2 cells was approved by the Brock University Bioscience Research Ethics Board (#17–347).

### Materials and chemicals

 Saos-2 cells were obtained from the ATCC (Manassas, VA, USA). Whole-leaf teas were purchased from a local tea shop and the same batch was used to ensure consistency. Ham's F12 medium and antibiotic/antimycotic were purchased from Lonza (Mississauga, ON, Canada), whereas fetal bovine serum (FBS), and trypsin-versene were purchased from GIBCO (Thermo Fisher Scientific, Waltham, MA, USA). All other chemicals were purchased from Sigma-Aldrich (Oakville, ON, Canada). Laboratory consumables were purchased from Sarstedt (Nümbrecht, Germany) and optical density (OD) measurements were determined using a BIO-TEK Synergy HT Multi-Detection Microplate Reader (Winooski, VT, USA).

### Preparation of tea samples

Loose-leaf tea (0.2 g) was steeped in water (5 mL) at the manufacturer's recommended temperature and duration (Table 1) to reflect how a consumer would steep their tea. The resulting tea preparations were filtered through a 0.2 μm filter to ensure there was no solid debris.

**Table 1.** Loose-Leaf Tea Characteristics and Steeping Conditions

Sample identification	Common name	Steep time (min)	Steep temperature (°C)	Scientific name (variety)	Type
WT	Bai Hao Yin Zhen	5	79	*Camellia sinensis* (L.) Kuntze	White tea
GT	Dragon well	3	79	*C. sinensis* (L.) Kuntze	Green tea
BT	English Breakfast	3	96	*C. sinensis* (L.) Kuntze	Black tea
GR	Green Rooibos	5	96	*Aspalathus linearis* (Burm.f.) R. Dahlgren	Herbal
RR	Red Rooibos	5	96	*A. linearis* (Burm.f.) R. Dahlgren	Herbal

### Determination of total polyphenol content and antioxidant capacity

Total polyphenol content (TPC) was determined according to the International Organization of Standardization (ISO 14502–1) with slight modifications as previously described.^12,21,22^ To determine the antioxidant capacity of each tea type, the ability to inhibit the free radical 2,2-diphenyl-1-picrylhydrazyl (DPPH) was measured as previously described.^12,21–23^ In brief, tea preparations were diluted to a concentration of 40 μg/mL, and 50 μL of diluted sample was added to 1.95 mL of 60 μM DPPH and incubated in the dark for 1 h. OD of the sample was measured at 517 nm.

### Cell culture

Saos-2 cells were treated as previously described with slight modifications.^12^ Cells were maintained at 37°C in 5% CO_2_ in media-1 (Ham's F12 medium supplemented with 10% FBS [v/v], 1% antibiotic/antimycotic [v/v], 2.0 mM L-glutamine, 28 mM HEPES buffer, and 1.0 mM CaCl_2_). To induce differentiation, cells were seeded in 24-well plates at a density of 1 × 10^4^ cells/well in media-2 (media 1 + 10 nM dexamethasone and 50 μg/mL ascorbic acid) for 8 days. After differentiation, media-3 (media-2 supplemented with 10 mM β-glycerophosphate and either vehicle control [dH_2_O] or one of the tea preparations [concentration of 1 μg/mL of GAE]) was added to initiate mineralization. This concentration of polyphenols from tea (1 μg/mL) is representative of what could likely be obtained through diet.^24^ All experiments were done using cells at passages between 50 and 60 and media were replenished every 2 or 3 days to ensure consistency.

### Mineralization and cellular activity

Cell mineralization was quantified using Alizarin Red S (ARS) staining as previously described previously.^12^ OD of the resulting mixture was then measured in triplicate at 550 nm. To measure the influence of tea preparations (concentration of 1 μg/mL of GAE) on cell activity, the 3-(4,5-dimethylthiazol-2-yl)-2,5-diphenyltetrazolium bromide (MTT) assay was performed as has been described.^12^ OD of the mixture was measured in triplicate at 570 nm with background at 690 nm.

### Quantitative reverse transcription PCR

Gene expression (*ALP*, *NPP1*, *OPN*, and *SOST*) was quantified following 5 days of mineralization as previously published.^12^ Extraction of total RNA was completed using the RNeasy© Plus Mini Kit (Qiagen: Hilden, Germany) according to the manufacturer's recommendations and the purity and quantity of collected RNA was determined using a NanoVue Plus system (Biochrom, Holliston, MA, USA). Reverse transcription of RNA to cDNA was completed using EcoDry double-primed premix (Clontech: Mountain View, CA, USA). All primers were synthesized by Integrated DNA Technologies (IDT: Coralville, IA, USA), and before testing were validated for efficiency and specificity in the used Saos-2 cell model. Samples were run in duplicate on a 96-well reaction plate and analyzed using the 2^−ΔΔCT^ method with GAPDH as a reference gene. For forward and reverse primer sequences see Table 2.

**Table 2.** Forward and Reverse Primer Sequences

Gene	Forward primer (5′→3′)	Reverse primer (5′→3′)	Accession number
*ALP*	CTT GTG CCT GGA CGGACCCT	TGG TGC ACC CCA AGA CCT GC	NM_000478.5
*NPP1*	CGC TGT TTC GAG AGA ACA TTT GGG A	AGT CGC CCT TGT CCT TGC AGT	NM_006208.2
*SOST*	CCA CCG GAG CTG GAG AAC AAC A	ATC GGT CAC GTA GCG GGT GAA G	NM_025237
*OPN*	TCA GCC AAA CGC CGA CCA AG	TGG AAG GGT CTG TGG GGC TAG GAG	AB469789.1
*GAPDH*	CCT GTT CGA CAG TCA GCC GCAT	TGG TGA CCA GGC GCC CAA TA	NM_002046.5

### Statistical analyses

One-way analysis of variance were conducted to assess differences in TPC, antioxidant activity, mineralization, cellular activity, and gene expression. If a significant difference (*P* < .05) was observed in any of the statistical analyses, Tukey's *post-hoc* test was performed to further identify where the specific differences were located. All statistical analyses were conducted using GraphPad Prism™ V5 (La Jolla, CA, USA). Results are reported as mean (TPC and antioxidant activity), mean % of control (mineralization and cellular activity), and mean fold change compared with control (gene expression) with errors reported as ±standard error of the mean.

## Results

### Total polyphenol content

**FIG. 1.**
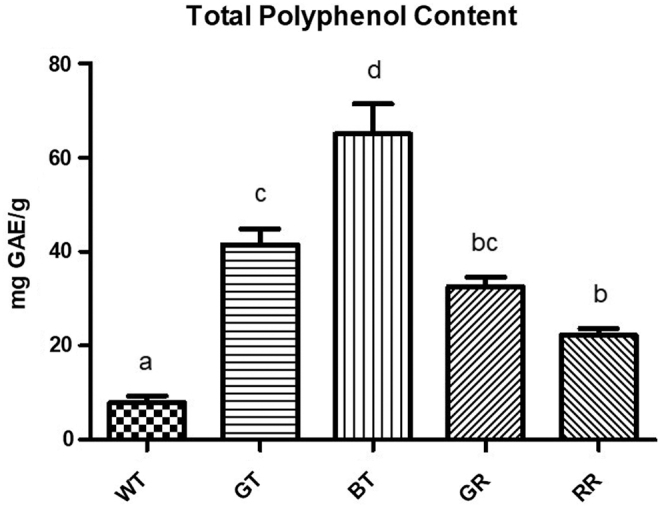
TPC of different types of tea after steeping. Error bars are ± SEM, *n* = 8 biological replicates. *Differing letters* indicate a significant difference (*P* < .05) between tea types. SEM, standard error of the mean; TPC, total polyphenol content.

After steeping loose-leaf tea at the recommended temperature and duration, TPC of tea types differed significantly (*P* < .05) (Fig. 1). BT had the greatest TPC followed by GT and GR. There were no significant differences in TPC between the two varieties of rooibos (GR and RR); whereas WT had the lowest TPC of all teas tested.

### Antioxidant capacity

**FIG. 2.**
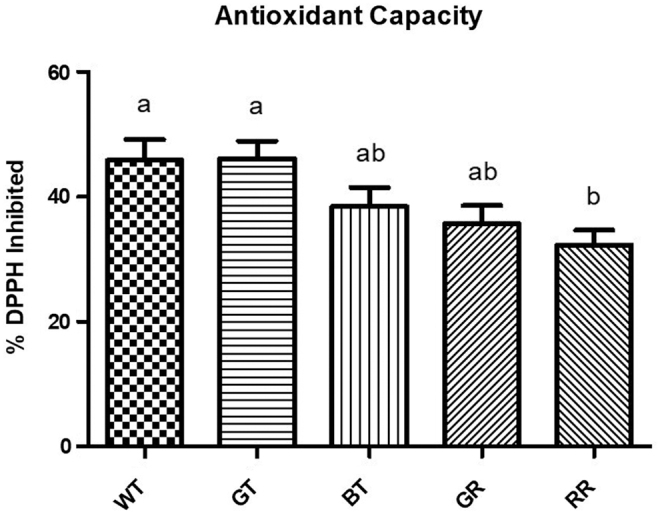
The ability of a normalized amount (1 μg/mL) of polyphenols from different tea types to inhibit the free radical DPPH. Error bars are ±SEM, *n* = 5 biological replicates. *Differing letters* indicate a significant difference (*P* < .05) between tea types. DPPH, 2,2-diphenyl-1-picrylhydrazyl.

Following normalization for polyphenol content (1 μg/mL of GAE), significant differences (*P* < .05) in the antioxidant capacity of tea types were observed (Fig. 2). WT and GT inhibited a significantly greater amount of DPPH when compared with RR, whereas BT and GT did not differ from the other tea types.

### Mineralization

**FIG. 3.**
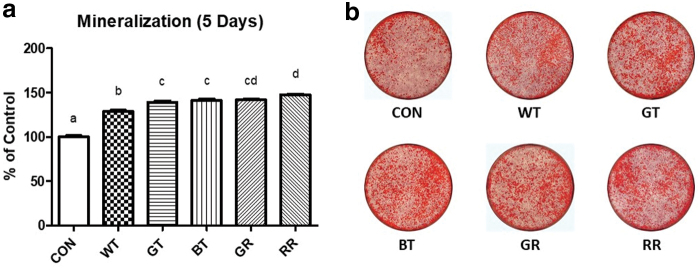
Effect of varying tea types on mineralization. **(a)** Mineralization following 5 days in the presence of different types of tea or a vehicle control. Error bars are ±SEM, *n* = 24 wells. *Different letters* indicate a significant (*P* < .05) difference between condition. **(b)** Representative images of mineralization after Alizarin Red stain. Color images are available online.

After 5 days of mineralization, the quantity of mineral produced was measured. Cells exposed to any tea type produced significantly greater (*P* < .05) quantities of mineral than control; WT produced the least mineral of all teas, whereas RR produced significantly more minerals than GT and BT (Fig. 3).

### Cell activity

**FIG. 4.**
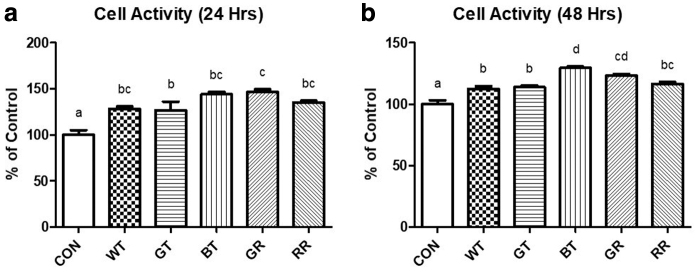
Cell activity measured through the formation of formazan crystals by the reduction of MTT following **(a)** 24 h, and **(b)** 48 h of exposure to varying tea types or a vehicle control. Errors are ±SEM, *n* = 15 wells. *Different letters* indicate a significant difference (*P* < .05) between conditions. MTT, 3-(4,5-dimethylthiazol-2-yl)-2,5-diphenyltetrazolium bromide.

After the addition of MTT (5 mg/mL), the resulting formation of formazan crystals were measured after 24 and 48 h of exposure to different tea types or a vehicle control. After 24 h, all tea types displayed increased cell activity compared with control (*P* < .05) (Fig. 4a). Among teas, BT, GR, and RR, elicited greater cell activity than WT. In comparison, after 48 h, elevated levels of cell activity persisted in cells exposed to tea, regardless of type, compared with control (*P* < .05); whereas differences were observed among tea types (Fig. 4b). More specifically, BT had greater cell activity than WT, GT, and RR; GR elicited greater cell activity than WT and GT.

### Quantification of gene expression

**FIG. 5.**
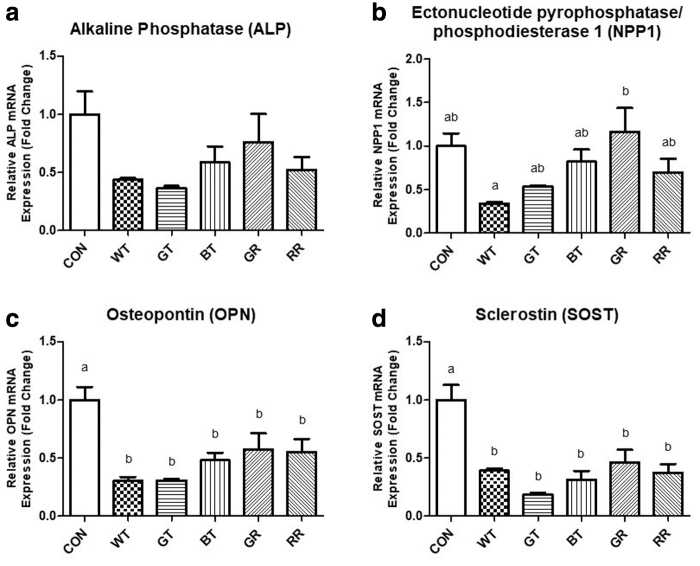
Gene expression following 5 days of mineralization for **(a)** *ALP*, **(b)** *NPP1*, **(c)** *OPN*, and **(d)** *SOST*. Error bars are ±SEM, *n* = 6 biological replicates. *Different letters* indicate a significant difference (*P* < .05) between conditions. *ALP*, alkaline phosphatase; *NPP1*, ectonucleotide pyrophosphatase/phosphodiesterase; *OPN*, osteopontin; *SOST*, sclerostin.

After 5 days of mineralization, the expression of several genes was measured. *ALP* expression did not differ between any of the groups (Fig. 5a), whereas there were only slight differences in *NPP1* expression with GR being greater than WT (Fig. 5b). *OPN* gene expression (Fig. 5c) and *SOST* (Fig. 5d) showed similar alterations with all tea types displaying reduced expression compared with control with no differences among tea types.

## Discussion

Findings from this study demonstrate that black, green, and both rooibos teas stimulated mineralization more than white tea, but all five tea types investigated significantly increased mineralization when compared with a vehicle control. This finding aligns with a previous study that directly compared black, green, and green rooibos tea and showed that all three increased mineralization compared with control, although no differences among these tea types was observed. These results provide support to the potential beneficial effects that tea may have on bone formation and possibly BMD.^25^ In the present study, the observed increase in mineralization for all tea types may be due to polyphenols in tea functioning as antioxidants and as a result reducing oxidative stress.^26–28^ Oxidative stress has been shown to negatively impact osteoblasts through reductions in both differentiation and mineralization.^29^ Non-tea polyphenols, such as astragalin and sciadopitysin, have been shown to increase osteoblast differentiation and mineralization through their ability to function as antioxidants and decrease oxidant stress.^30,31^ Interestingly, despite a normalized quantity of polyphenols from each tea, there were differences in mineralization between the tea types (GT/BT/GR/RR > WT, GT = BT = GR, RR > BT/GT) suggesting that the unique polyphenol profile of each tea may be responsible for the observed differences in mineralization between tea types. In addition, the mineralization data do not follow the antioxidant capacities of the teas. WT had one of the greatest antioxidant capacities of the teas studied, however; it elicited the lowest amount of mineralization of all teas. This suggests that mechanisms in addition to antioxidant activity may also be occurring to cause the changes in mineralization. It is also possible that these other mechanisms may be the result of other healthy components found in tea (*i.e.*, trace minerals and vitamins).

Cell activity was increased for all tea types. It has been well documented that osteoblasts will undergo apoptosis and a reduction in activity in response to oxidative stress.^32–34^ Several studies have also demonstrated that antioxidants from plant sources, such as apigenin, curcumin, and aucubin, are able to increase osteoblast viability in many osteoblast cell models, including; Saos-2, MC3T3–E1, and MG-63.^35–37^ These findings provide support that polyphenols found in the tea likely acted as antioxidants to reduce oxidative stress thus increasing activity and mineralization. More specifically, using the same cell model, researchers have observed both elevated cell activity and mineralization in response to tea (black, green, and rooibos).^11–13^

*ALP* and *NPP1* are genes encoding for proteins directly related to phosphate balance and mineralization. More specifically, alkaline phosphatase (ALP) increases free phosphate, which can be used to produce mineral; whereas ectonucleotide pyrophosphatase/phosphodiesterase (NPP1) increases pyrophosphate, which inhibits mineralization.^38^ In spite of the increased mineralization from cells exposed to tea, there were no alterations in gene expression of either *ALP* or *NPP1* following 5 days of mineralization. It is possible that despite there being no significant changes in mRNA expression of *ALP* or *NPP1*, there may be extensive post-translational modification to these proteins.^39^ Previous studies involving tea and osteoblasts have generally seen increases in both ALP protein quantity and activity.^11,14^ In osteoblasts, osteopontin (OPN) functions to inhibit the mineralization process.^40^ OPN has been shown to be regulated by oxidative stress, with greater amounts of stress eliciting greater expression of *OPN*.^41–43^ After 5 days of mineralization, the expression of *OPN* was significantly reduced for cells receiving any tea compared with cells that received a vehicle. Polyphenols from the tea likely functioned as antioxidants reducing oxidant stress in the cells and as a result OPN as well; providing a mechanistic change that may partially explain the observed changes in mineralization and aligns with findings in the literature.^12^ Sclerostin (SOST) is another well-documented inhibitor of mineralization functioning to inhibit the Wnt pathway and osteoblast differentiation, proliferation, and activity.^44–46^ Our findings demonstrate that tea, regardless of type, reduced expression of *SOST* and inhibition of mineralization.

In the present study, polyphenol content was normalized for each tea to compare the different polyphenol profiles rather than the amount of polyphenols present. However, it is likely that the effectiveness of tea to promote mineralization is dependent on both the quantity and specific profile of polyphenols within a tea and more work is needed to clarify this aspect. For example, despite rooibos teas eliciting similar levels of mineralization as black tea, due to the ∼2-fold greater quantity of polyphenols found in black tea, twice as much rooibos tea will need to be consumed to receive similar levels of polyphenols. Although there were observed differences in mineralization among tea types, this may not translate to clinical differences in humans, measured as differences in BMD or risk of fracture. The key message for a consumer is that all tea types increased mineralization, and while future study is needed to confirm *in vivo* effects, consuming any of the teas studied may benefit bone health.

In conclusion, our findings demonstrate that supplementation of Saos-2 cells with tea increased mineralization regardless of tea type; however, there are differences among tea types with both rooibos teas and black tea eliciting greater mineralization than white tea. Future studies should try to elucidate mechanisms other than antioxidant effects that are playing a role in causing the observed increase in mineralization and the biological significance of the findings *in vivo*.
